# Antithrombotic Management after Successful Catheter Ablation for Atrial Fibrillation: Meta-analysis of the ALONE-AF and OCEAN Trials

**DOI:** 10.1055/a-2916-8912

**Published:** 2026-08-21

**Authors:** Wen-Han Cheng, Yi-Hsin Chan, Ling Kuo, Jo-Nan Liao, Chih-Min Liu, Boyoug Joung, Tze-Fan Chao

**Affiliations:** 1 Division of CardiologyDepartment of Internal Medicine 218818 National Yang Ming Chiao Tung University HospitalYilanTaiwan ProvinceTaiwan; 2 46615 Cardiovascular Center, Taipei Veterans General HospitalTaipei CityTaipei CityTaiwan; 3 34882 Institute of Clinical Medicine and Cardiovascular Research Center, National Yang Ming Chiao Tung UniversityTaipei CityTaipei CityTaiwan; 4 Department of Cardiovascular 38014 Chang Gung Memorial Hospital LinkouTaoyuanTaoyuan CityTaiwan; 5 56081 College of Medicine, Chang Gung UniversityTaoyuanTaoyuan CityTaiwan; 6 56081 School of Traditional Chinese Medicine, Chang Gung UniversityTaoyuanTaoyuan CityTaiwan; 7 38014 Microscopy Core Laboratory, Chang Gung Memorial Hospital LinkouTaoyuanTaoyuan CityTaiwan; 8 Division of Cardiology 37991 Severance Hospital, Yonsei University College of MedicineSeodaemun-guSeoulKorea (the Republic of)

**Keywords:** ablation, anticoagulation, atrial fibrillation, bleeding, stroke

## Abstract

**Background and Aims** Current guidelines advocate for indefinite direct oral anticoagulant (DOAC) therapy after successful catheter ablation for atrial fibrillation (AF), based on the CHA _2_ DS _2_ -VASc score. This strategy is challenged by the inherent bleeding risk of DOAC versus the potentially reduced stroke risk post-ablation. We aimed to compare long-term antithrombotic strategies in patients with durable freedom from AF recurrence.

**Methods** We conducted a meta-analysis of the ALONE-AF and OCEAN trials enrolling 2124 patients with no documented AF recurrence for ≥1 year after ablation. The trials compared DOAC continuation versus DOAC discontinuation. We calculated pooled risk ratios (RRs) with 95% confidence intervals (CIs) for primary efficacy (thromboembolic events/all-cause mortality) and safety (bleeding events) outcomes.

**Results** The meta-analysis found no statistically significant difference between DOAC continuation and DOAC discontinuation for all stroke or systemic embolism (RR 1.36; 95% CI: 0.16–11.38), transient ischemic attack (RR 0.20; 95% CI: 0.03–1.15), myocardial infarction (RR 0.08; 95% CI: 0.00–1.37), and all-cause mortality (RR 1.43; 95% CI: 0.55–3.74). In contrast, DOAC continuation was associated with a significantly higher risk of major bleeding (RR 3.07; 95% CI: 1.05–8.96) and clinically relevant non-major bleeding (RR 2.47; 95% CI: 1.01–6.00). Subgroup analysis confirmed consistent treatment effects.

**Conclusion** In patients who achieve durable freedom from AF recurrence for at least 1 year following catheter ablation, the absolute risk of thromboembolic events was very low. Strategies involving the discontinuation of DOACs were associated with a statistically lower risk of bleeding without significant risk of stroke.

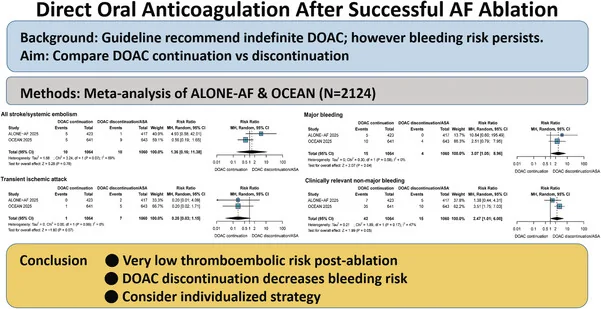

## Introduction

Catheter ablation has become an established rhythm-control therapy for atrial fibrillation (AF), yet its impact on long-term thromboembolic risk remains unclear. 1 2 3 Current international guidelines largely advocate for the continuation of direct oral anticoagulant (DOAC) therapy indefinitely after successful ablation based on the CHA _2_ DS _2_ -VASc score. 1 2

The rationale for continuation of DOAC is the potential for subclinical or recurrent AF and the difficulty in predicting which patients will remain protected from stroke. 1 2 However, DOAC therapy carries an inherent risk of bleeding, which may outweigh the marginal benefit of stroke prevention in a population with a potentially reduced post-ablation thromboembolic risk.

Recently, two large randomized controlled trials (RCTs) have tested the efficacy and safety of discontinuation of DOAC in post-ablation patients who had remained free of documented AF recurrence for more than 12 months. The ALONE-AF (Anticoagulation One Year after Ablation of Atrial Fibrillation in Patients with Atrial Fibrillation) trial evaluated whether patients who had no documented AF recurrence for at least 1 year after ablation could safely discontinue DOAC, and whether doing so would reduce adverse events compared with continued DOAC therapy. 4 The OCEAN (Optimal Anticoagulation for Enhanced Risk Patients Post-Catheter Ablation for Atrial Fibrillation) trial investigated whether rivaroxaban provides better protection than aspirin (ASA) against stroke, systemic embolism, and covert embolic stroke in patients with stroke risk factors following successful catheter ablation for AF. 5 In brief, both trials revealed that among post-ablation patients with no documented AF recurrence, stopping DOAC was comparable regarding the composite risk of stroke, systemic embolism, and major bleeding relative to continued DOAC therapy. 4 5

The objective of this meta-analysis was to pool and compare the findings of these two recent RCTs that have specifically addressed the long-term antithrombotic management strategy in patients with a history of AF who have achieved durable freedom from AF recurrence following catheter ablation.

## Methods

The current meta-analysis did not require institutional research ethics board approval. All primary data supporting the findings of this study are presented within the manuscript.

### Eligibility Criteria

We searched for RCTs enrolling adults (≥18 years) with a history of AF who had no documented recurrence of AF for at least 12 months after catheter ablation, and that compared antithrombotic strategies such as continuation versus discontinuation of DOAC.

### Search Methods

**Fig. 1:**
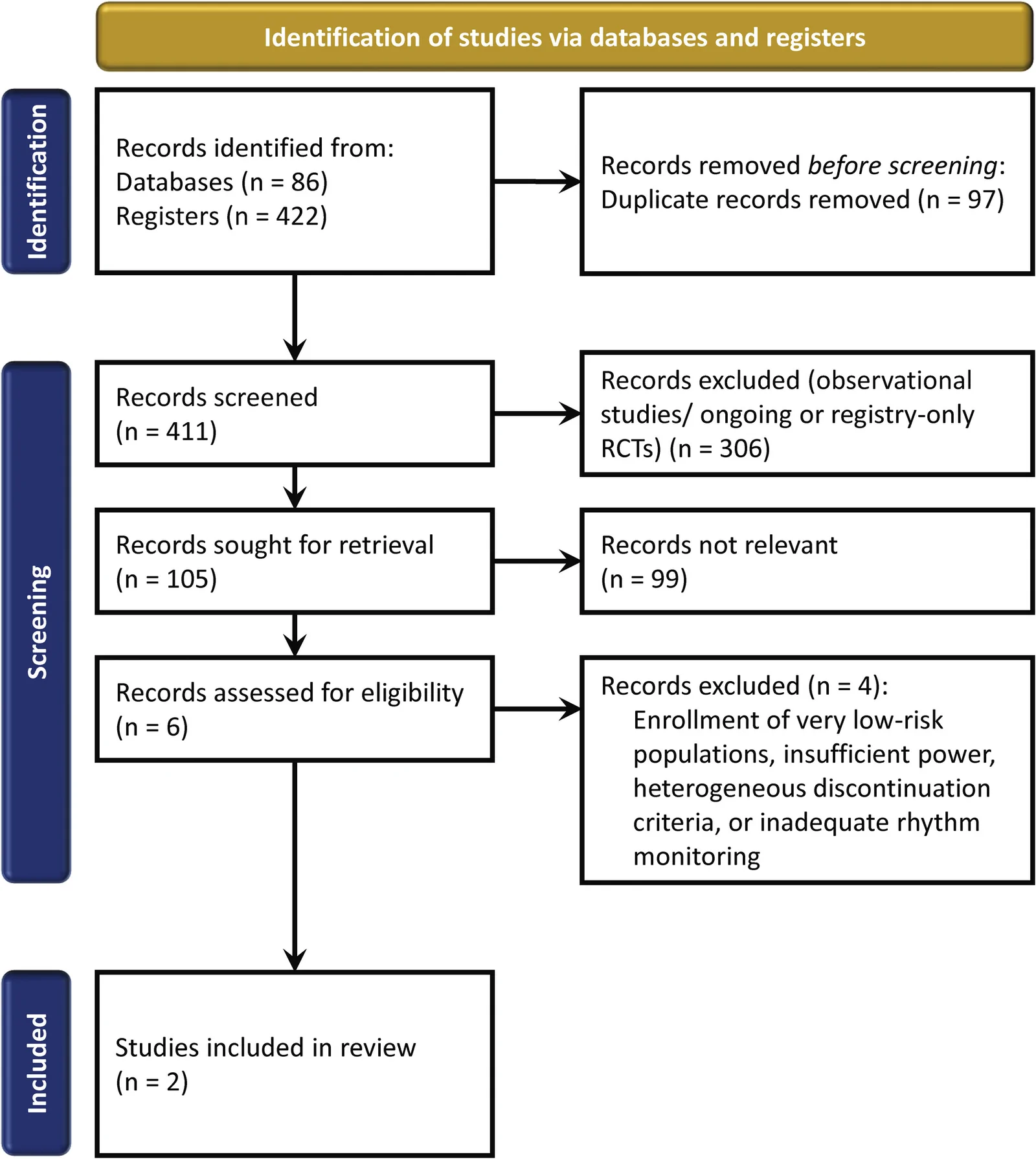
Study selection diagram.

We searched PubMed, MEDLINE, and the Cochrane Central Register of Controlled Trials from 2000 through December 2025 using a combination of keywords and Medical Subject Headings to identify studies related to post-AF ablation management and oral anticoagulants (OAC). We used a validated filter for RCTs 6 ( Fig. 1 ).

### Selection of Studies

Two reviewers (Y.H.C. and T.F.C.) independently and in duplicate screened titles and abstracts. Full-text articles were retrieved for all studies considered potentially relevant by either reviewer. Subsequently, two authors (W.H.C. and Y.H.C.) independently assessed the full-text articles against the predefined eligibility criteria. Any disagreements were resolved through discussion and consensus.

### Data Extraction

Data were extracted from the key summary findings of the included trials. The extracted data included: patient population characteristics (sample size, mean age, CHA _2_ DS _2_ -VASc score), study interventions, definition of successful ablation, follow-up duration, and key outcomes.

### Outcomes

The primary outcomes of this meta-analysis were the major composite efficacy and safety endpoints reported in the each trial, including all stroke or systemic embolism, transient ischemic attack (TIA), myocardial infarction (MI), all-cause mortality, major bleeding, intracranial hemorrhage (ICH), gastrointestinal (GI) bleeding, other major bleeding, and clinically relevant non-major bleeding.

The subgroup analysis further examined the pooled primary outcomes defined in each trial based on age strata, sex, AF type before ablation, CHA _2_ DS _2_ -VASc score, and cardiovascular comorbidities. In ALONE-AF, the primary outcome was the first occurrence of a composite of stroke, systemic embolism, and major bleeding at 2 years; in OCEAN, the primary outcome was a composite of stroke, systemic embolism, or newly detected covert embolic stroke at 3 years.

### Risk of Bias

We evaluated risk of bias using the Cochrane RevMan version 5.4.1. Two reviewers independently rated each study as having “low,” “high,” or “uncertain” risk of bias across seven domains: random sequence generation, allocation concealment, blinding of participants and personnel, blinding of outcome assessment, incomplete outcome data, selective reporting, and other bias. We classified the overall risk of bias as low when all domains were rated low, as some concerns when at least one domain raised concerns but none were rated high, and as high when one or more domains were judged to be at high risk of bias ( **Supplementary Fig. S1** (online only)).

### Quality Assessment

We assessed the quality of evidence using the Grading of Recommendations Assessment, Development and Evaluation approach. 7 To determine our confidence in the effect estimates, we considered the risk of bias in individual studies, the directness of the evidence, the precision of effect estimates for each clinical outcome, the degree of heterogeneity, and the potential for publication bias 8 ( **Supplementary Table S1** (online only)).

### Statistical Analysis

To evaluate associations between treatment groups and the outcomes of interest, we calculated risk ratios (RRs) with corresponding 95% confidence intervals (CIs) for binary outcomes and mean differences with 95% CIs for continuous outcomes. Pooled estimates were generated using inverse-variance-weighted random-effects models with the DerSimonian and Laird estimator for between-study variance (τ ^2^ ). A continuity correction was applied when necessary for individual study calculations and for the pooled analyses. Heterogeneity was assessed using the *I* ^2^ statistic and τ ^2^ , with *I* ^2^ > 50% or a τ ^2^ *p* -value < 0.05 indicating substantial heterogeneity. Meta-analytic results were visualized with forest plots. All analyses were performed using RStudio, Version 1.2.1335. 9

## Results

### Selection of Included Trials

A total of 2124 patients were randomized across the two trials. The ALONE-AF trial randomized 840 patients and compared the discontinuation of DOAC therapy ( *n* = 417) with the continuation of DOAC therapy ( *n* = 423). The OCEAN trial randomized 1284 patients and compared rivaroxaban (15 mg daily, *n* = 641) with ASA (70–120 mg daily, *n* = 643). Both trials defined successful ablation as no documented recurrence of atrial arrhythmia (AF, atrial flutter, or atrial tachycardia ≥30 s) for at least 1 year prior to randomization. The characteristics of these RCTs are summarized in Table 1 and **Supplementary Tables S2–S5** (online only).

**Table 1:** Characteristics of enrolled trials.

Trials	ALONE-AF trial		OCEAN-AF trial	
Total patients	*N* = 840		*N* = 1284	
Treatment group	DOAC continued *N* = 423	DOAC discontinued *N* = 417	DOAC continued *N* = 641	DOAC discontinued *N* = 643
Age (years)	65 ± 8	63 ± 8	66.3 ± 7.1	66.3 ± 7.6
Male sex	310 (73.3%)	321 (77.0%)	458 (71.5%)	459 (71.4%)
Time from ablation to randomization	2.3 years (IQR: 1.2–4.5)	2.5 years (IQR: 1.4–5.2)	16.4 months (IQR: 13.4–25.2)	16.5 months (IQR: 13.6–25.2)
Type of AF				
Paroxysmal	292 (69.0%)	276 (66.2%)	431 (67.2%)	421 (65.5%)
Persistent	131 (31.0%)	141 (33.8%)	204 (31.8%)	212 (33.0%)
Long-standing persistent	—	—	6 (0.9%)	10 (1.6%)
Number of ablations				
1	—	—	484 (75.5%)	504 (78.4%)
2	—	—	133 (20.7%)	105 (16.3%)
≥3	—	—	24 (3.7%)	34 (5.3%)
CHA _2_ DS _2_ -VASc score				
Mean	2 (IQR: 1–3)	2 (IQR: 1–3)	2.2 ± 1.1	2.2 ± 1.1
Score = 1	120 (28.4%)	125 (30.0%)	194 (30.3%)	196 (30.5%)
Score = 2	172 (40.7%)	165 (39.6%)	241 (37.6%)	243 (37.8%)
Score = 3	83 (19.6%)	83 (19.9%)	138 (21.5%)	127 (19.8%)
Score ≥ 4	48 (11.3%)	42 (10.1%)	68 (10.6%)	77 (12.0%)
HAS-BLED score	2 (IQR: 1–3)	2 (IQR: 1–3)	1.4 ± 0.9	1.3 ± 0.8
Comorbidities				
Hypertension	291 (68.8%)	293 (70.3%)	442 (69.0%)	434 (67.5%)
Diabetes mellitus	90 (21.3%)	68 (16.3%)	96 (15.0%)	77 (12.0%)
Heart failure	62 (14.7%)	66 (15.8%)	—	—
Dyslipidemia	125 (29.6%)	102 (24.5%)	—	—
Coronary artery disease	—	—	74 (11.5%)	68 (10.6%)
Myocardial infarction	8 (1.9%)	4 (1.0%)	—	—
Ischemic cardiomyopathy	—	—	9 (1.4%)	6 (0.9%)
Non-ischemic cardiomyopathy	—	—	15 (2.3%)	20 (3.1%)
Peripheral artery/vascular disease	10 (2.4%)	3 (0.7%)	9 (1.4%)	9 (1.4%)
Chronic kidney disease	3 (0.7%)	10 (2.4%)	—	—
Stroke or TIA	24 (5.7%)	23 (5.5%)	—	—
Type of stroke				
Ischemic	—	—	7 (1.1%)	17 (2.6%)
Hemorrhagic	—	—	0	2 (0.3%)
Uncertain	—	—	3 (0.5%)	6 (0.9%)
TIA	—	—	18 (2.8%)	25 (3.9%)
Current drinking	107 (25.3%)	121 (29.0%)	431 (70.6%)	438 (68.1%)
Current smoking	36 (8.5%)	54 (12.9%)	25 (3.9%)	35 (5.4%)
Blood pressure				
Systolic (mmHg)	127 (IQR: 118–138)	128 (IQR: 120–138)	134.4 ± 16.2	135.4 ± 16.7
Diastolic (mmHg)	75 (IQR: 68–82)	77 (IQR: 70–84)	79.4 ± 9.3	79.3 ± 10.2
Body mass index	25.0 (IQR: 23.1–27.1)	25.3 (IQR: 23.6–27.7)	28.2 ± 5.0	27.7 ± 4.6
Creatinine clearance (mL/min)	85.4 ± 14.1	85.9 ± 13.1	94.2 ± 31.8	93.6 ± 29.7
Echocardiography				
Left atrial dimension (mm)	40 (IQR: 37–44)	40 (IQR: 37–43)	40.7 ± 16.0	40.4 ± 19.2
Left ventricle ejection fraction (%)	62 (IQR: 57–66)	61 (IQR: 57–66)	61.8 ± 7.1	61.5 ± 7.3
LV function normal	—	—	476 (99.2%)	453 (97.8%)
LV function abnormal	—	—	4 (0.8%)	10 (2.2%)
E/e' ratio	9.2 (IQR: 7.7–11.9)	8.9 (IQR: 7.5–11.0)	—	—

Abbreviations: AF, atrial fibrillation; DOAC, direct oral anticoagulant; IQR, interquartile range; LV, left ventricle; TIA, transient ischemic attack.

### Primary Efficacy Outcomes

**Fig. 2:**
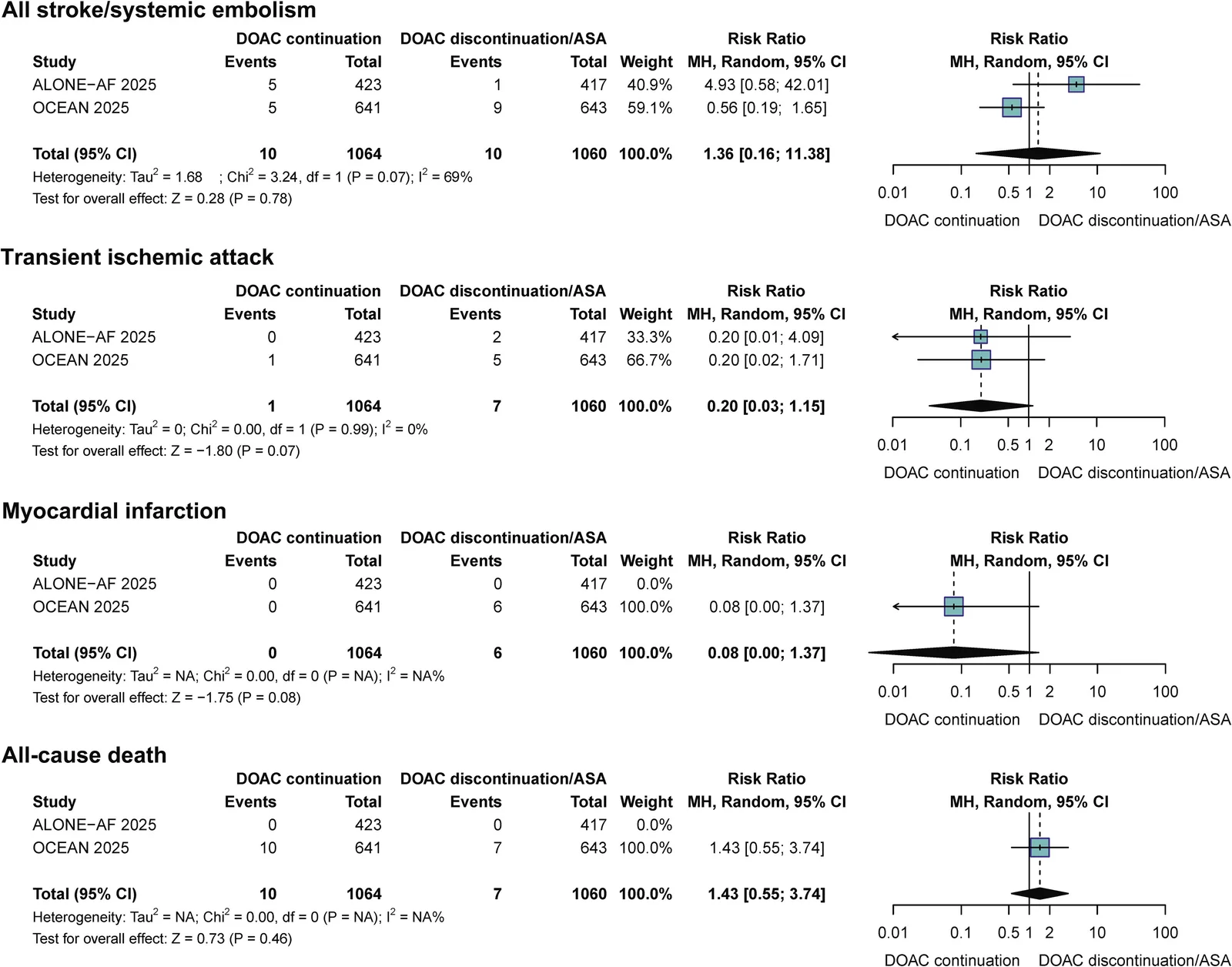
Primary efficacy outcomes comparing direct oral anticoagulant (DOAC) continuation versus discontinuation. ASA, aspirin; CI, confidence interval.

The meta-analysis comparing DOAC continuation to DOAC discontinuation included a total of 2124 participants and revealed no statistically significant difference between the two strategies for any of the primary outcomes ( Fig. 2 and Table 2 ; **Supplementary Fig. S2** and **Supplementary Table S6** (online only)). For all stroke or systemic embolism, 10 events occurred in each group, resulting in a pooled RR of 1.36 (95% CI: 0.16–11.38). For TIA, the DOAC continuation group had 1 event compared with 7 events in the DOAC discontinuation group, yielding an RR of 0.20 (95% CI: 0.03–1.15). For MI, the DOAC continuation group recorded 0 events compared with 6 events in the DOAC discontinuation group, resulting in an RR of 0.08 (95% CI: 0.00–1.37). For all-cause mortality, the DOAC continuation group had 10 events compared with 7 events in the DOAC discontinuation group, yielding an RR of 1.43 (95% CI: 0.55–3.74).

**Table 2:** Reported outcomes of enrolled trials.

Study and outcome	DOAC group (Continued/Rivaroxaban)	No DOAC group (Discontinued/Aspirin)			
ALONE-AF trial, 2025	Continued DOAC ( *n* = 423)	Discontinued DOAC ( *n* = 417)	Relative risk (95% CI) (Calculated)	Absolute difference (95% CI)	Number need to treat/harm
**Primary outcomes**					
Composite: stroke, systemic embolism, major bleeding	8 (2.2%)	1 (0.3%)	7.89 (1.00 to 62.1)	+1.9 (+0.3 to +3.5)	53 (favoring no DOAC)
**Efficacy outcomes**					
All stroke	5 (1.4%)	1 (0.3%)	4.93 (0.58 to 42.0)	+1.1 (−0.3 to +2.4)	
Ischemic stroke	3 (0.8%)	1 (0.3%)	2.96 (0.31 to 28.3)	+0.5 (−0.6 to +1.6)	
Hemorrhagic stroke	2 (0.6%)	0 (0%)	4.93 (0.24 to 102.4)	+0.6 (−0.2 to 1.3)	
Systemic embolism	0 (0%)	0 (0%)	—	—	
TIA	0 (0%)	2 (0.6%)	0.20 (0.01 to 4.09)	−0.6 (−1.3 to 0.2)	
Myocardial infarction	0 (0%)	0 (0%)	—	—	
Hospitalization due to any cause	38 (10.0%)	30 (8.3%)	1.25 (0.80 to 1.96)	+1.7 (−2.5 to 5.8)	
All-cause mortality	0 (0%)	0 (0%)	—	—	
**Safety outcomes**					
Major bleeding	5 (1.4%)	0 (0%)	10.84 (0.60 to 195.5)	+1.4 (+0.2 to +2.6)	
Intracranial bleeding	2 (0.6%)	0 (0%)	4.93 (0.24 to 102.4)	+0.6 (−0.2 to 1.3)	
Gastrointestinal bleeding	2 (0.5%)	0 (0%)	4.93 (0.24 to 102.4)	+0.5 (−0.2 to 1.3)	
Other major bleeding	1 (0.3%)	0 (0%)	2.96 (0.12 to 72.4)	+0.3 (−0.3 to 0.8)	
Fatal bleeding	0 (0%)	0 (0%)	—	—	
Clinically relevant non-major bleeding	7 (1.9%)	5 (1.4%)	1.38 (0.44 to 4.31)	+0.5 (−1.4 to +2.4)	
**OCEAN trial, 2025**	** Rivaroxaban ( *n* = 641) **	** Aspirin ( *n* = 643) **	**Relative risk (95% CI)**	**Absolute difference (95% CI) (calculated)**	**Number need to treat/harm**
**Primary efficacy outcomes**					
Composite: stroke, systemic embolism, new covert embolic stroke	5 (0.8%)	9 (1.4%)	0.56 (0.19 to 1.65)	−0.6 (−1.8 to 0.5)	167
All stroke	5 (0.8%)	7 (1.1%)	0.72 (0.23 to 2.25)	−0.3 (−1.4 to 0.7)	333
Systemic embolism	0 (0.0%)	0 (0.0%)	—	—	
New covert embolic stroke	0 (0%)	2 (0.3%)	0.20 (0.01 to 4.17)	−0.3 (−0.7 to 0.1)	333
TIA	1 (0.2%)	5 (0.8%)	0.20 (0.02 to 1.71)	−0.6 (−1.4 to 0.1)	167
All-cause mortality	10 (1.6%)	7 (1.1%)	1.43 (0.55 to 3.74)	+0.5 (−0.8 to 1.7)	200
**Primary safety outcomes**					
Composite: fatal or major bleeding	10 (1.6%)	4 (0.6%)	2.51 (0.79 to 7.95)	+0.9 (−0.2 to 2.1)	111
Major bleeding	10 (1.6%)	4 (0.6%)	2.51 (0.79 to 7.95)	+0.9 (−0.2 to 2.1)	111
Intracranial bleeding	5 (0.8%)	1 (0.2%)	5.02 (0.59 to 42.81)	+0.6 (−0.1 to 1.4)	167
Gastrointestinal bleeding	3 (0.5%)	2 (0.3%)	1.50 (0.25 to 8.97)	+0.2 (−0.5 to 0.8)	500
Other major bleeding	2 (0.3%)	1 (0.2%)	2.01 (0.18 to 22.07)	+0.1 (−0.4 to 0.7)	1000
Fatal bleeding	0 (0%)	0 (0%)	—	—	
Minor bleeding	74 (11.5%)	20 (3.1%)	3.71 (2.29 to 6.01)	+8.4 (5.6 to 11.3)	12
Clinically relevant non-major bleeding	35 (5.5%)	10 (1.6%)	3.51 (1.75 to 7.03)	+3.9 (1.9 to 5.9)	26
Major or minor bleeding	83 (12.9%)	23 (3.6%)	3.62 (2.31 to 5.67)	+9.4 (6.4 to 12.3)	11

Abbreviations: CI, confidence interval; DOAC, direct oral anticoagulant; TIA, transient ischemic attack.

### Primary Safety Outcomes

**Fig. 3:**
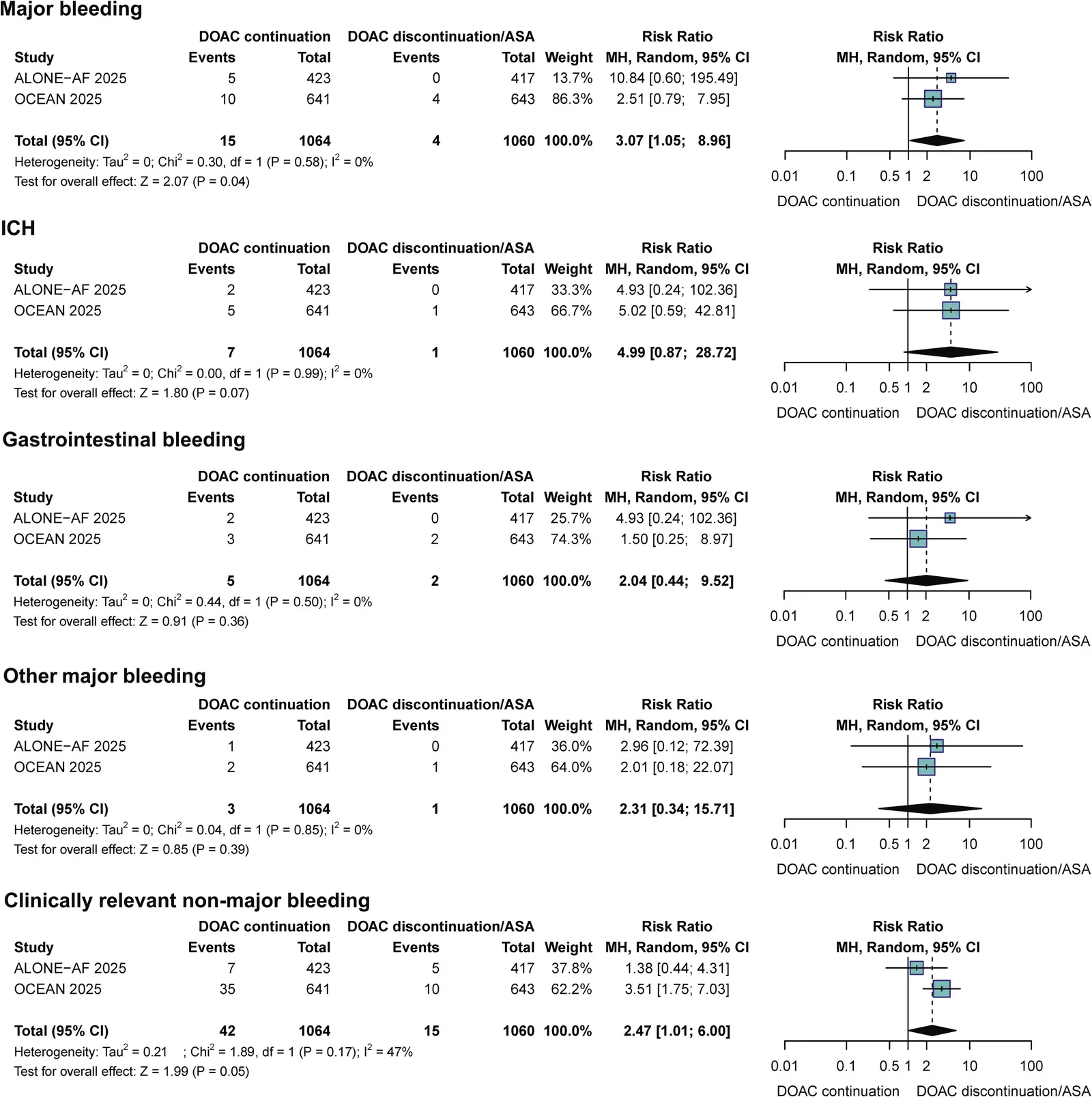
Primary safety outcomes comparing direct oral anticoagulant (DOAC) continuation versus discontinuation. ASA, aspirin; CI, confidence interval; ICH, intracranial hemorrhage.

The meta-analysis evaluating bleeding risks showed statistically significant difference between DOAC continuation and DOAC discontinuation for major bleeding and clinically relevant non-major bleeding ( Fig. 3 and Table 2 ; **Supplementary Fig. S3** and **Supplementary Table S6** (online only)). For major bleeding, there were 15 events in the DOAC continuation group compared with 4 events in the DOAC discontinuation group, resulting in a pooled RR of 3.07 (95% CI: 1.05–8.96). For clinically relevant non-major bleeding, 42 events occurred in the DOAC continuation group compared with 15 events in the DOAC discontinuation group, with a pooled RR of 2.47 (95% CI: 1.01–6.00).

On the contrary, the meta-analysis assessing other bleeding risks showed no statistically significant difference between DOAC continuation and DOAC discontinuation ( Fig. 3 and Table 2 ; **Supplementary Fig. S3** and **Supplementary Table S6** (online only)). For ICH, 7 events occurred in the DOAC continuation group compared with 1 event in the DOAC discontinuation group, yielding an RR of 4.99 (95% CI: 0.87–28.72). In GI bleeding, 5 events were reported in the DOAC continuation group compared with 2 events in the DOAC discontinuation group, with an RR of 2.04 (95% CI: 0.44–9.52). Other major bleeding had 3 events in the DOAC continuation group versus 1 event in the DOAC discontinuation group, resulting in an RR of 2.31 (95% CI: 0.34–15.71).

### Subgroup Analysis of Pooled Primary Outcomes in Each Trial

**Fig. 4:**
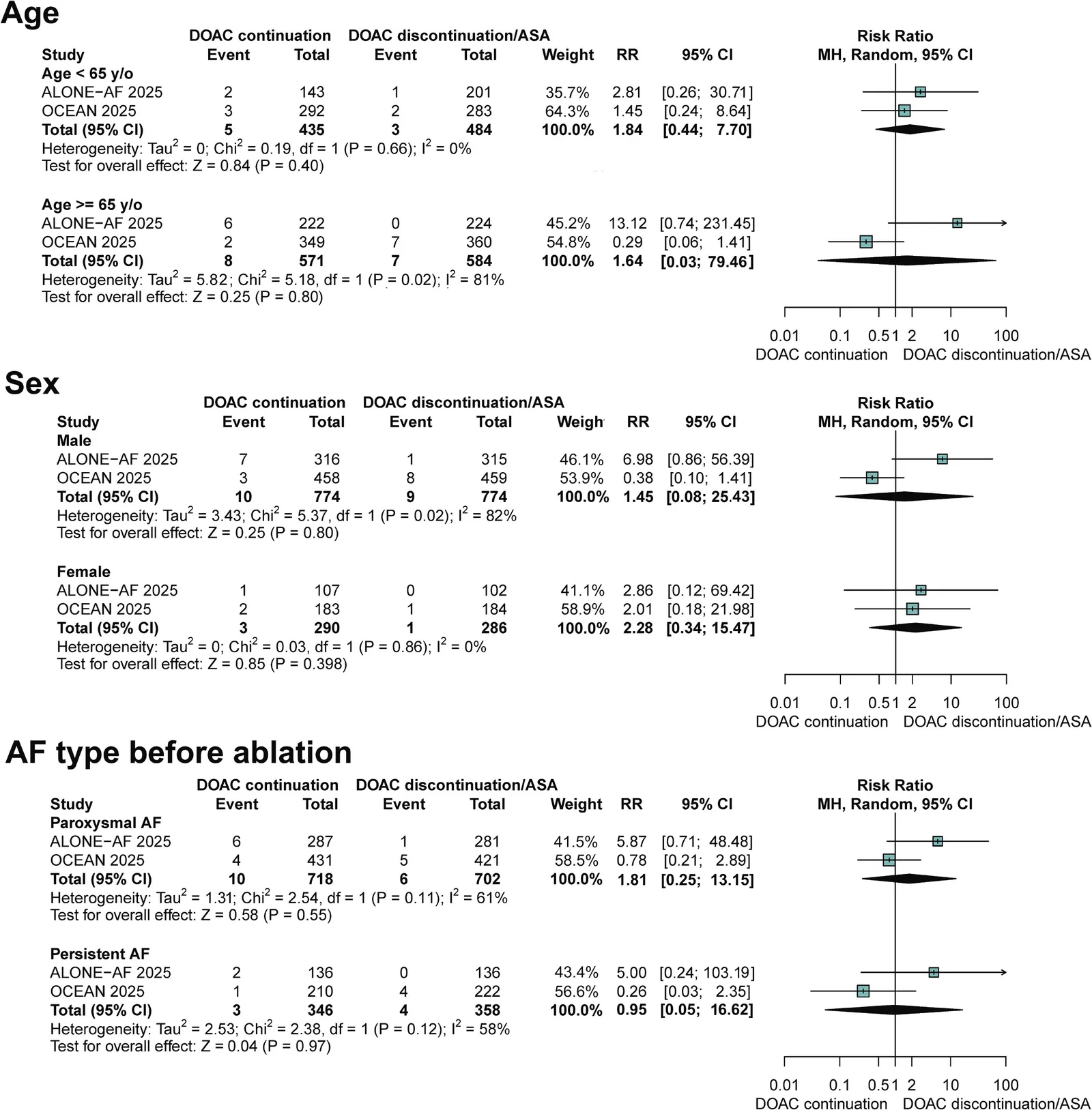
Subgroup analysis of pooled primary outcomes in each trial comparing direct oral anticoagulant (DOAC) continuation versus discontinuation based on age strata, sex, and atrial fibrillation (AF) type before ablation. ASA, aspirin; CI, confidence interval.

**Fig. 5:**
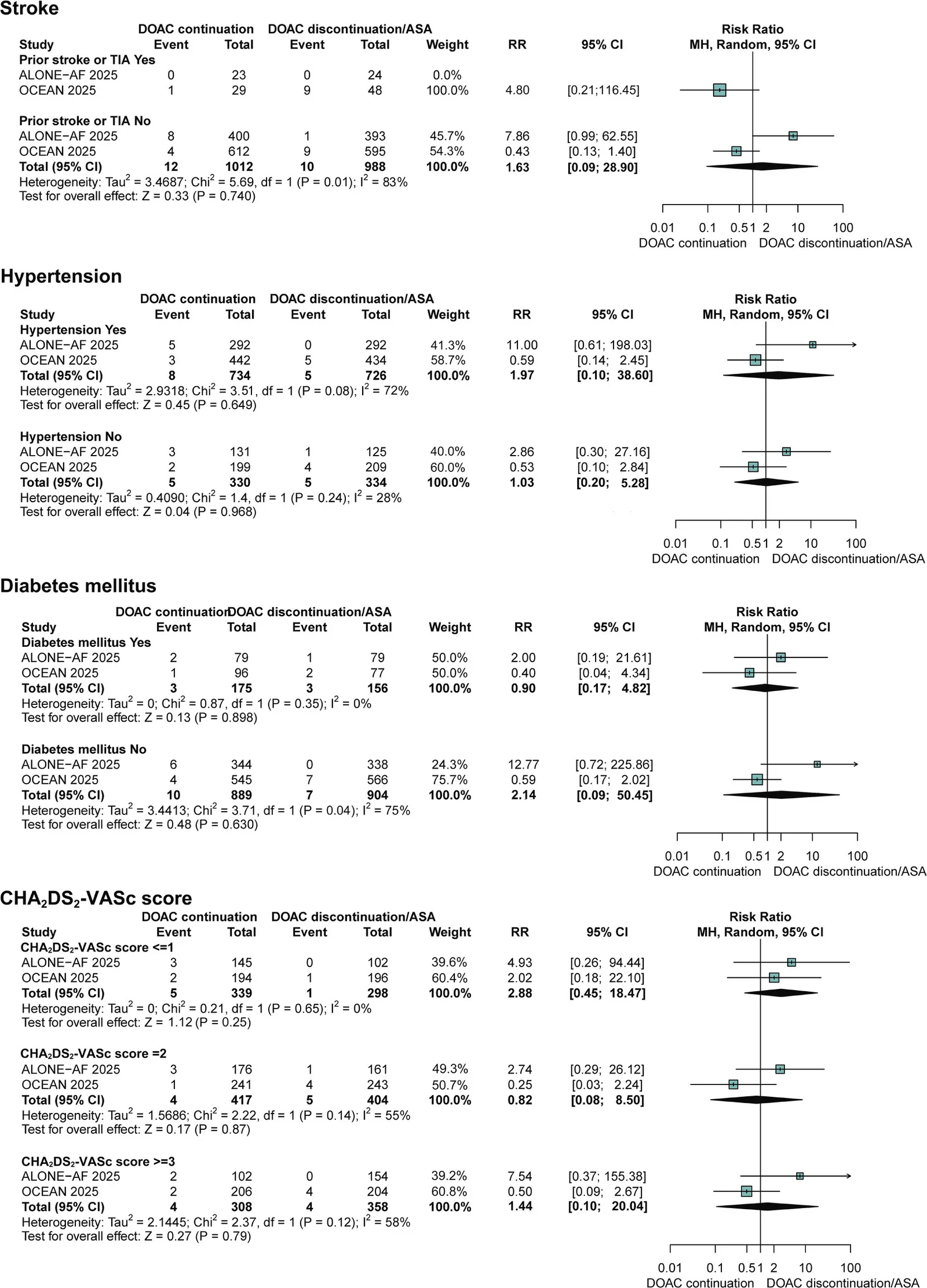
Subgroup analysis of pooled primary outcomes in each trial comparing direct oral anticoagulant (DOAC) continuation versus discontinuation based on cardiovascular comorbidities and CHA _2_ DS _2_ -VASc score. ASA, aspirin; CI, confidence interval.

The subgroup analysis investigated the pooled primary outcomes in each trial comparing DOAC continuation versus DOAC discontinuation. The treatment effect did not exhibit significant heterogeneity across the examined patient characteristics ( Figs. 4 and 5 ). The meta-analysis demonstrated non-significant RRs and their 95% CIs for across all subgroups, including age (<65 years: RR 1.84 [0.44–7.68]; ≥65 years: RR 1.64 [0.03–79.46]), sex (male: RR 1.45 [0.08–26.43]; female: RR 2.28 [0.34–15.47]), AF type before ablation (paroxysmal: RR 1.81 [0.25–13.15]; persistent: RR 0.95 [0.05–16.62]) ( Fig. 4 ), cardiovascular comorbidities such as history of stroke/TIA, hypertension, and diabetes mellitus: all crossed the line of no effect (RR = 1.0), and CHA _2_ DS _2_ -VASc scores: all crossed the line of no effect (RR = 1.0) ( Fig. 5 ).

## Discussion

In this up-to-date and comprehensive systematic review and meta-analysis, we found that discontinuing DOAC did not increase the risk of thromboembolic events compared with continued DOAC therapy among patients who had remained free of documented AF recurrence for at least 1 year. Across all randomized patients and a broad spectrum of stroke-risk profiles, the absolute incidence of thromboembolic events was remarkably low in both treatment strategies, including all stroke or systemic embolism, TIA, MI, and all-cause mortality. In contrast, bleeding outcomes demonstrated clear differences: continuation of DOAC was consistently associated with a significantly higher risk of major bleeding and clinically relevant non-major bleeding. Moreover, subgroup analysis showed consistency of thromboembolic events throughout different conditions.

The current meta-analysis revealed overall low absolute rate of thromboembolic events in both groups, especially when compared with historical non-ablation AF cohorts of similar CHA _2_ DS _2_ -VASc score. 10 In prior studies of medically managed AF, annualized stroke rates typically ranged from 1.5 to 4%, depending on CHA _2_ DS _2_ -VASc score and DOAC use. 10 By contrast, in both ALONE-AF and OCEAN, 4 5 annualized rates of all stroke or systemic embolism fell below 1% per year in both the continuation and discontinuation groups. This phenomenon likely reflects the impact of effective rhythm control after ablation. Catheter ablation reduces AF burden substantially, often eliminating sustained arrhythmia episodes for years. 11 Given the well-documented association between AF burden and stroke risk, 8 12 13 a marked reduction in AF burden may diminish the risk for thromboembolism, thereby narrowing the relative benefit of anticoagulation in a subset of post-ablation patients. Nonetheless, the majority of participants in both trials had moderate stroke risk, with a mean CHA _2_ DS _2_ -VASc score of 2–2.2. 4 5 While the current meta-analysis findings suggest that DOAC discontinuation strategies may be safe for such patients, caution is warranted for individuals at very high thromboembolic risk, who were underrepresented or excluded. We propose our perspective on post-ablation stroke prevention strategies in patients without evidence of recurrence ( Table 3 ).

**Table 3:** Expert opinion on post-ablation stroke prevention strategies in patients without evidence of recurrence.

	Patient groups	Stroke prevention strategy
Low stroke risk	CHA _2_ DS _2_ -VASc score 0–1 (M) or 1–2 (F)	No OACs
Intermediate stroke risk	CHA _2_ DS _2_ -VASc score 2 (M) or 3 (F)	No OACs or OACs based on shared decision-making
High stroke risk	CHA _2_ DS _2_ -VASc score ≥3 (M) or ≥4 (F) or Prior stroke/Transient ischemic attack or Age ≥75 years or Vascular diseases	OACs

Abbreviation: OAC, oral anticoagulants.

Moreover, the current meta-analysis demonstrated that while thromboembolic events were similar between the DOAC continuation and discontinuation groups, bleeding risks were not. The continuation of DOAC therapy was associated with nearly a 3-fold increase in major bleeding and clinically relevant non-major bleeding. The OCEAN trial further showed a higher incidence of minor bleeding and clinically relevant non-major bleeding with rivaroxaban compared with ASA. 5 These findings are aligned with the established safety profiles of DOAC therapies, which have been consistently shown to increase the risk of bleeding compared with control/antiplatelet therapy. 14 15 Therefore, the decision to continue DOAC in this population must weigh a low event rate of ischemic complications against the risk of bleeding. Given that major bleeding was significantly lower in the OAC discontinuation group than in the continuation group in the ALONE-AF trial, possibly due to the low rate of ASA use, ASA should not be used when OAC is discontinued. 4

In addition, heterogeneity across patient subgroups in the current meta-analysis was minimal. Across age strata, sex, AF type before ablation, CHA _2_ DS _2_ -VASc score, and cardiovascular comorbidities, treatment effects were consistent, showing that no specific subgroup appeared to derive clear thromboembolic benefit from ongoing DOAC despite freedom from AF recurrence. Although certain point estimates favored DOAC continuation for ischemic endpoints, all subgroup confidence intervals were wide due to low event rates and limited statistical power, making definitive subgroup conclusions impossible. Nevertheless, this consistent pattern suggests that the potential de-escalation of DOAC therapy may be broadly applicable across a range of post-ablation patient profiles if no AF recurrence is detected.

Lastly, the current meta-analysis findings must be interpreted in the context of how AF recurrence was assessed in these trials. Both RCTs required documented AF freedom based on intermittent Holter monitoring and symptom-triggered evaluations. 4 5 Intensive continuous rhythm monitoring such as implantable loop recorders or wearable devices was not used. 16 Therefore, asymptomatic or short-duration AF episodes may have gone undetected. 17 The use of intermittent Holter monitoring may explain why thromboembolic events, although rare, were not completely eliminated in any treatment arm. Future RCTs incorporating intensive continuous rhythm surveillance may help refine patient selection for safe DOAC discontinuation and clarify whether even minimal subclinical AF should preclude cessation of anticoagulation after successful catheter ablation. 18

## Limitations

This meta-analysis has several limitations. First, because ALONE-AF compared DOAC continuation versus DOAC discontinuation (no antithrombotic therapy), whereas OCEAN compared rivaroxaban versus ASA, treating ASA as the “discontinuation” arm conflates antiplatelet therapy with true DOAC cessation. While ASA is not equivalent to the complete absence of antithrombotic therapy, its use for stroke prevention in AF is no longer recommended by current international guidelines. 1 2 Consequently, our meta-analysis sought to clarify the distinct impact of DOAC cessation in this specific group of patients. Moreover, the included trials differed in follow-up duration, with ALONE-AF reporting approximately 2.5 years of follow-up and OCEAN approximately 1.4 years. These differences may have influenced event detection and contributed to between-study heterogeneity, particularly given the very low incidence of thromboembolic outcomes observed in both trials.

## Conclusion

In patients who achieved durable freedom from AF recurrence following catheter ablation, thromboembolic event rates were low irrespective of treatment strategy. Although less intensive antithrombotic approaches were associated with reduced bleeding, the available randomized evidence remains insufficient to definitively establish the comparative efficacy of continued versus discontinued anticoagulation for stroke prevention.

**What is Known About this Topic?**

- Guidelines currently favor indefinite direct oral anticoagulant (DOAC) therapy after atrial fibrillation (AF) ablation according to CHA _2_ DS _2_ -VASc score. This approach reflects concern that stroke risk may persist despite apparent procedural success.
- Long-term DOAC therapy carries an ongoing bleeding hazard. This may offset benefit in patients whose post-ablation stroke risk is substantially reduced.
- Individual randomized trials have suggested that OAC discontinuation may be feasible in select post-ablation patients.

**What Does this Paper Add?**

- In patients free from documented AF recurrence for ≥1 year after ablation, thromboembolic risk was very low. Continued DOAC therapy did not significantly reduce ischemic events or mortality compared with discontinuation strategies.
- DOAC continuation significantly increased major bleeding and clinically relevant non-major bleeding. The excess bleeding risk was not accompanied by a clear ischemic benefit.
- This meta-analysis provides pooled randomized evidence supporting a more individualized approach to long-term anticoagulation after successful AF ablation.

## Data Availability

All primary data supporting the findings of this study are presented within the manuscript.

## Supplementary Material

Ergänzendes Material Supplementary Material
