# Supplementary material for: Antithrombotic Management after Successful Catheter Ablation for Atrial Fibrillation: Meta-analysis of the ALONE-AF and OCEAN Trials
Source: TH Open. 2026 Aug 21;10:a29168912. doi: 10.1055/a-2916-8912 (PMC13552201; doi:10.1055/a-2916-8912)
Supplement: Supplementary file 1 [file 10-1055-a-2916-8912_29343404.pdf]

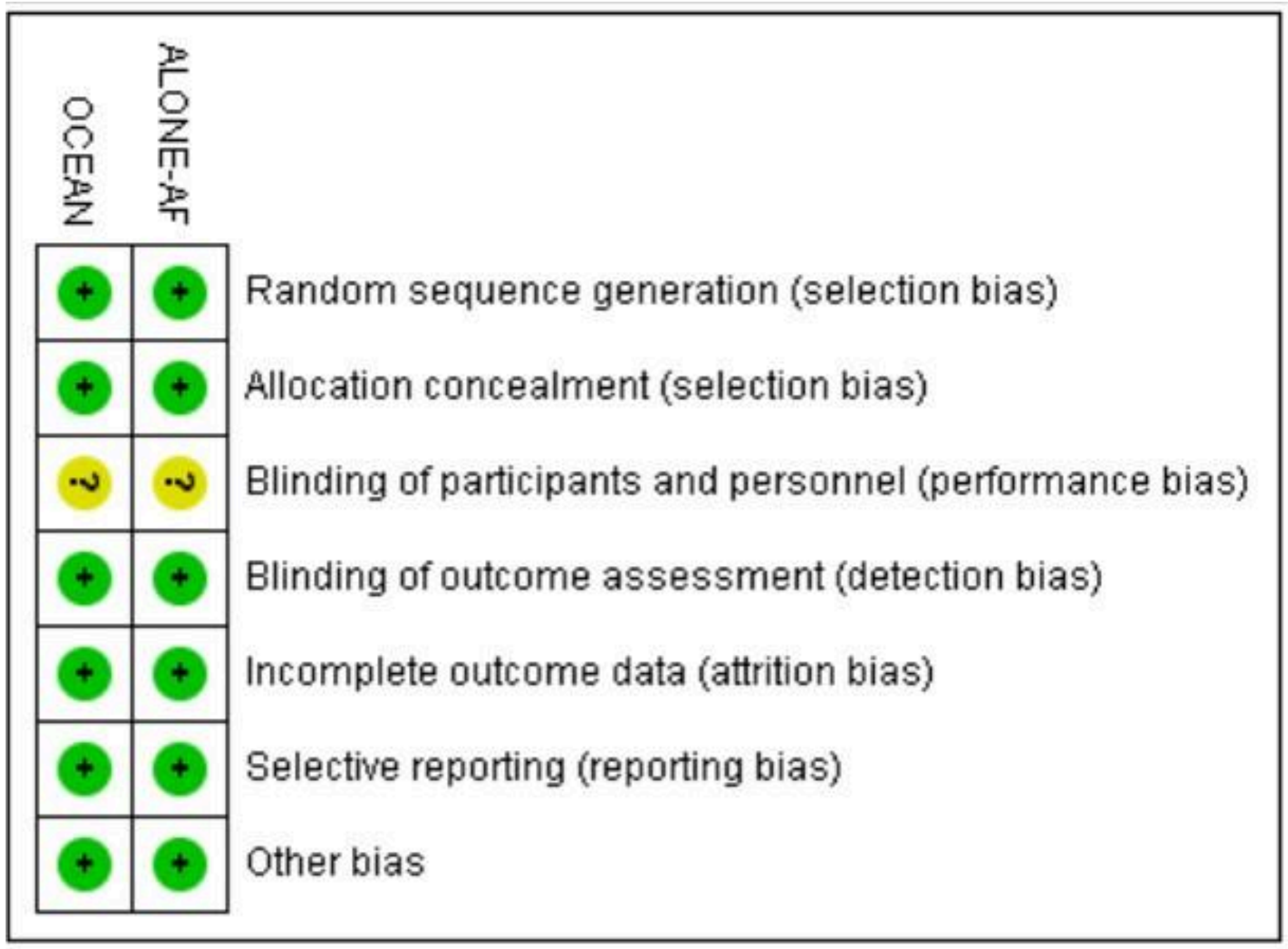

Supplementary Fig. S1. Risk of bias.

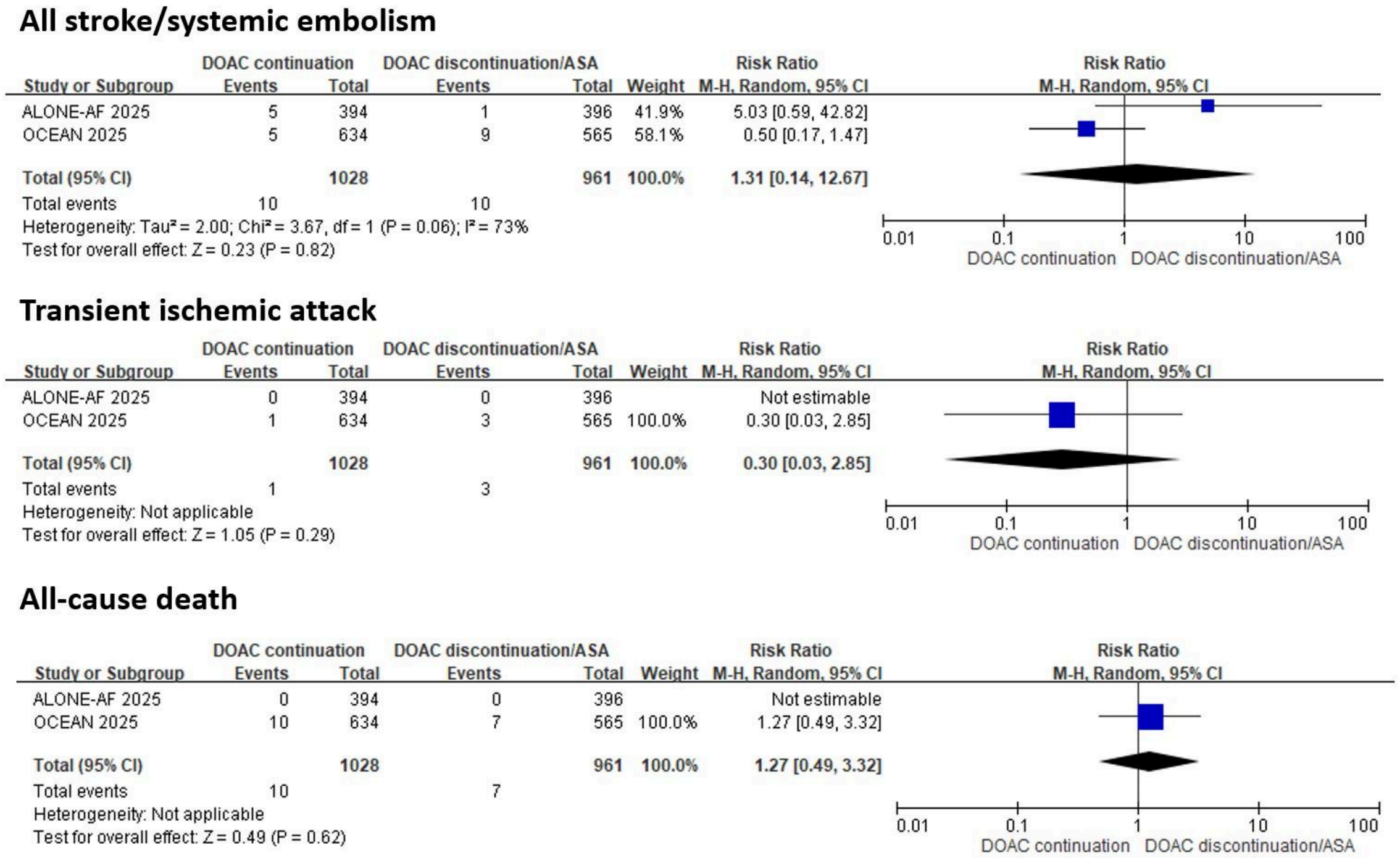

Supplementary Fig. S2. Primary efficacy outcomes comparing DOAC continuation versus discontinuation (per protocol population).

ASA: aspirin, CI: confidence interval, DOAC: direct oral anticoagulant

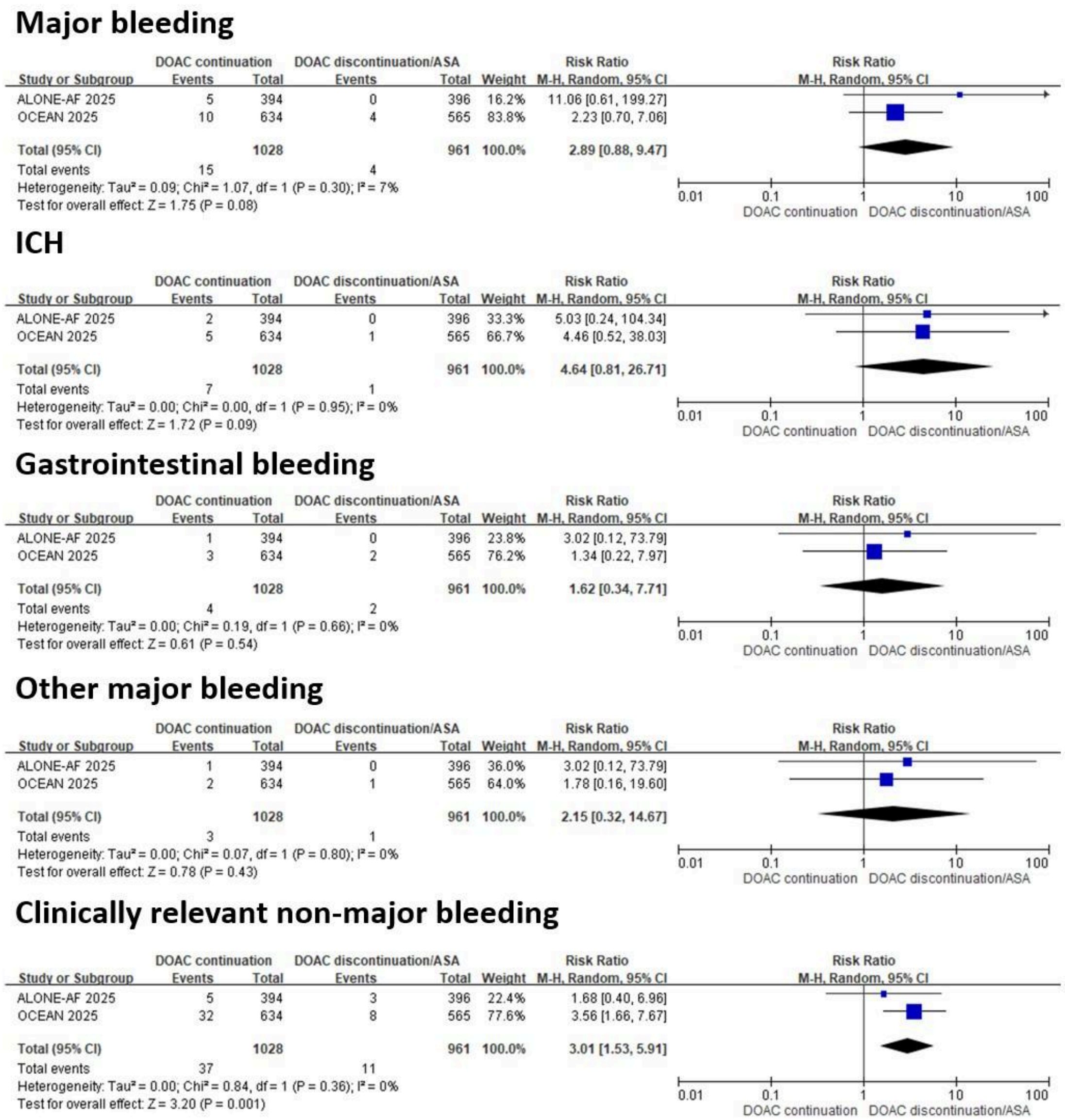

Supplementary Fig. S3. Primary safety outcomes comparing DOAC continuation versus discontinuation (per protocol population).

ASA: aspirin, CI: confidence interval, DOAC: direct oral anticoagulant, ICH: intracranial hemorrhage

Supplemental Materials

Supplementary Table S1. GRADE Assessment of the enrolled trials.

Outcome	Number of studies	Study design	Risk of bias	Inconsistency	Indirectness	Imprecision	Risk Ratio (95% CI)	Certainty
**All stroke or systemic embolism**	2	Randomized trials	Not serious	Not serious	Not serious	Very serious	1.36 (0.16-11.38)	⊕⊕◯◯ **Low**
**Transient ischemic attack**	2	Randomized trials	Not serious	Not serious	Not serious	Very serious	0.20 (0.03-1.15)	⊕⊕◯◯ **Low**
**Myocardial infarction**	2	Randomized trials	Not serious	Not serious	Not serious	Very serious	0.08 (0.00-1.37)	⊕⊕◯◯ **Low**
**All-cause mortality**	2	Randomized trials	Not serious	Not serious	Not serious	Serious	1.43 (0.55-3.74)	⊕⊕⊕◯ **Moderate**
**Major bleeding**	2	Randomized trials	Not serious	Not serious	Not serious	Serious	2.92 (1.06-8.07)	⊕⊕⊕◯ **Moderate**
**Intracranial hemorrhage**	2	Randomized trials	Not serious	Not serious	Not serious	Very serious	4.99 (0.87-28.72)	⊕⊕◯◯ **Low**
**Gastrointestinal**	2	Randomized	Not	Not serious	Not serious	Very serious	2.04 (0.44-	⊕⊕◯◯ **Low**

**bleeding**		trials	serious				9.52)	
**Other major bleeding**	2	Randomized trials	Not serious	Not serious	Not serious	Very serious	2.31 (0.34-15.71)	⊕⊕◯◯ **Low**
**Clinically relevant non-major bleeding**	2	Randomized trials	Not serious	Not serious§	Not serious	Not serious	2.47 (1.01-6.00)	⊕⊕⊕⊕ **High**

CI: confidence interval

Supplementary Table S2. Inclusion and exclusion criteria for the enrolled trials.

**Criteria Category**	**ALONE-AF Trial**	**OCEAN Trial**
**Target Population**	Patients with a history of AF who underwent first-time catheter ablation and had no recurrence of atrial arrhythmia for at least 12 months.	Patients who had undergone successful catheter ablation for nonvalvular AF at least 1 year before enrollment.
**Age**	19 to 80 years.	Excluded if >85 years of age.
**Stroke Risk Score *(CHA_2DS_2-VASc)***	CHA_2DS_2-VASc score ≥1 (male) or ≥ 2 (female)	CHA_2DS_2-VASc score ≥1 (male) or ≥ 2 (female) or patients where vascular disease was the risk factor.
**Definition of "No Recurrence"**	Absence of AF, atrial flutter, or atrial tachycardia lasting 30 seconds on monitoring.	No clinical evidence of arrhythmia and no atrial arrhythmia lasting > 30 seconds on monitoring.
**Monitoring Requirements Prior to Enrollment**	• At least two 24-72 hour Holter/ECG recordings conducted >3 months post-ablation.<br>• One mandatory recording within 2 months preceding enrollment.	• One 24-hour Holter between 2-6 months post-ablation.<br>• One 24-hour Holter >6 months post-ablation.<br>• One 48-hour Holter within 2 months preceding enrollment.
**Renal Exclusion**	Serum creatinine 3.5 mg/dL OR Creatinine clearance < 30 mL/min.	Creatinine clearance < 30 mL/min.
**Cardiovascular Exclusions**	• Mechanical prosthetic valve or moderate-to-severe mitral stenosis.<br>• Deep vein thrombosis requiring anticoagulation.<br>• Significant structural heart disease (e.g., moderate-to-severe mitral regurgitation, DCM, HCM).	• Valvular AF (rheumatic mitral valve disease or mechanical valve).<br>• Disabling stroke within the past year or any stroke within 14 days.<br>• Known intracranial vascular anomaly.
**Other Exclusions**	• Significant liver disease (AST/ALT >3x upper limit of normal).<br>• Active malignancy.	• Contraindication to anticoagulant or antiplatelet therapy.<br>• Hypercoagulability disorder.

	• Life expectancy < 1 year.<br>• Pregnancy or breast-feeding.	• Contraindication to MRI (e.g., incompatible devices, aneurysm clips).

AF: atrial fibrillation, DCM: dilated cardiomyopathy, HCM: hypertrophic cardiomyopathy, MRI: magnetic resonance imaging

Supplementary Table S3. Comparison of antithrombotic/antiplatelet therapy after randomization.

Feature	ALONE-AF Trial	OCEAN Trial
**Study Design Strategy**	• Discontinuation vs. Continuation<br>• Patients randomized to stop OAC or continue their current OAC.	• Active Comparator (Rivaroxaban vs. Aspirin)<br>• Patients randomized to a specific reduced-dose OAC (Rivaroxaban) or Aspirin.
**OAC Group Regimen**	• Apixaban: 78.0% (mostly 5 mg BID)<br>• Rivaroxaban: 16.0% (mostly 15-20 mg QD)<br>• Edoxaban: 0.7%<br>• Dabigatran: 0.0%<br>• Dosing: 85.6% received full dose; 14.4% received reduced dose.	• Rivaroxaban: Assigned dose of 15 mg QD.
**Comparator Group Regimen**	No Anticoagulant<br>• Patients discontinued all OACs.<br>• Antiplatelet therapy was generally discouraged unless clinically indicated (e.g., for PCI).	Aspirin<br>• Dose: 70 to 120 mg daily (depending on local availability).<br>• Patients intolerant to aspirin received no antiplatelet therapy.
**Antiplatelet Usage**	• No OAC Group: 8.6% received antiplatelets (7.4% Aspirin, 1.2% Clopidogrel).<br>• OAC Group: 5.0% received antiplatelets (4.7% Aspirin, 0.2% Clopidogrel).	• Aspirin Group: 100% assigned (except intolerance).<br>• Rivaroxaban Group: Protocol provided guidance for temporary dual antiplatelet therapy in cases of PCI/ACS (e.g., limited to 1–6 months),.
**Protocol Deviations/Crossovers**	• No OAC Group: 12 patients (2.9%) were prescribed OACs during follow-up (e.g., due to TIA or physician/patient preference).<br>• OAC Group: 12 patients (2.8%) discontinued OACs.	• Rivaroxaban Group: 34 patients received an alternative OAC due to adverse effects.<br>• Aspirin Group: 7 patients received no antiplatelet therapy due to adverse effects.

ACS: acute coronary syndrome, OAC: oral anticoagulant, PCI: percutaneous coronary intervention, TIA: transient ischemic attack

Supplementary Table S4. Comparison of AF Ablation Details in enrolled trials.

Feature	ALONE-AF Trial	OCEAN Trial
**Ablation history criteria**	Patients enrolled after their first-time catheter ablation without recurrence for at least 12 months.	Patients enrolled after one or more successful catheter ablations at least 1 year prior.
**Number of prior ablations**	1 (Implied by "first-time" inclusion criteria)	1 Ablation: 75.5%-78.4%<br>2 Ablations: 16.3%-20.7%<br>3 Ablations: 3.7%-5.3%
**Time from ablation to randomization**	Median: ~2.4 years (29-31 months reported in per-protocol baseline)	Median: ~1.4 years (16.4-16.5 months)
**Ablation modality**	Radiofrequency: 85% – 87%<br>Cryoballoon: 13% – 15%	Not reported
**Lesion sets performed**	Pulmonary Vein Isolation (PVI): 100%<br>Cavotricuspid Isthmus: 12%-14%<br>Linear Lesions (Roof/Ant/Post): 1%-3%<br>Complex Fractionated Electrograms: < 1.5%	Not reported
**Definition of "successful" ablation**	Absence of AF, atrial flutter, or atrial tachycardia lasting 30 seconds on Holter/ECG recordings.	No clinical evidence of arrhythmia and no atrial arrhythmia lasting >30 seconds on monitoring.
**Recurrence management (during Trial)**	Patients undergoing repeat AF ablation during the study were censored at the time of the event.	Patients undergoing repeat AF ablation during the study were withdrawn from the trial.

AF: atrial fibrillation

TH Open | DOI 10.1055/a-2916-8912 | © The Author(s).

Supplementary Table S5. Data regarding AF recurrence and management.

**Feature**	**ALONE-AF Trial**	**OCEAN Trial**
**Time since ablation**	3.6 years (mean) prior to randomization	1.4 years (16.4 months median) prior to randomization
**Definition of recurrence**	Any documented episode of AF, atrial flutter, or atrial tachycardia lasting 30 seconds.	Clinically apparent recurrence. The protocol categorized events as "Brief" (6 min), "Moderate" (6 min-24 hrs), or "Long" (>24 hrs).
**Monitoring strategy**	Structured Protocol:<br>Mandated 24- to 72-hour Holter monitoring at least every 6 months, plus routine ECGs at every visit. Additional monitoring if symptomatic.<br>12-lead ECG (65–84% of cases); Regular Holter (13–27% of cases); Wearables/Event Recorders (<8% of cases).	Standard of Care:<br>No mandated rhythm monitoring. Testing was performed only "as clinically indicated" or driven by symptoms.
**Recurrence rate**	~9.1% at 2 years (40/417 No OAC; 37/423 OAC).	~20.1% at 3 years (124/643 Aspirin; 134/641 Rivaroxaban).
**Median time to recurrence**	12.0 months after randomization (IQR: 6.0–17.4 months).	Not explicitly reported; follow-up duration was 3 years.
**Trial status upon recurrence**	Censored (Trial Exit):<br>Patients with confirmed recurrence were censored at the time of the event for the primary analysis.	Continued (Remain in Arm):<br>Investigators were encouraged to keep patients in their randomized arm. Withdrawal occurred only if the patient underwent repeat catheter ablation (5.5% of patients).
**Clinical management of recurrence**	Restart Anticoagulation:<br>Anticoagulation was reinitiated (in the No OAC group) or continued based on the patient's thromboembolic risk.	Maintain Therapy & Restore Rhythm:<br>The protocol advised maintaining the randomized therapy (Aspirin or Rivaroxaban) and using antiarrhythmic drugs or cardioversion to restore sinus rhythm. Cardioversion: Temporary OAC allowed (3 weeks prior, 4 weeks post). Brief/Moderate Recurrence: Maintain randomized therapy.

AF: atrial fibrillation, ECG: electrocardiography, OAC: oral anticoagulant

Supplementary Table S6. Reported outcomes of enrolled trials (per protocol population).

Study & Outcome	OAC Group (Continue/Rivaroxaban)	No OAC Group (Discontinue/Aspirin)		
**ALONE-AF TRIAL, 2025**	**Continued OAC (n=394)**	**Discontinued OAC (n=396)**	**Relative Risk (95% CI) (Calculated)**	**Absolute Difference (95% CI)**
**Primary Outcome**				
**Composite: Stroke, systemic embolism, major bleeding**	8 (2.3%)	1 (0.3%)	8.04 [1.01 to 64.0]	+2.0 [0.3 to 3.7]
**Efficacy Outcomes**				
Stroke (Total)	5 (1.5%)	1 (0.3%)	5.03 [0.59 to 42.8]	+1.1 [-0.3 to 2.6]
Ischemic Stroke	3 (0.9%)	1 (0.3%)	3.02 [0.31 to 28.9]	+0.5 [-0.6 to 1.7]
Hemorrhagic Stroke	2 (0.6%)	0 (0%)	5.03 [0.24 to 104.3]	+0.6 [-0.2 to 1.4]
Systemic Embolism	0 (0%)	0 (0%)	—	—
TIA	0 (0%)	0 (0%)	—	—
Myocardial Infarction	0 (0%)	0 (0%)	—	—
Hospitalization due to any cause	38 (10.6%)	26 (7.6%)	1.47 [0.91 to 2.37]	+3.0 [-1.2 to 7.3]
All-cause mortality	0 (0%)	0 (0%)	—	—
**Safety Outcomes**				
**Major Bleeding**	**5 (1.5%)**	**0 (0%)**	**11.06 [0.61 to 199.3]**	**+1.5 [+0.2 to +2.7]**
Intracranial Bleeding	2 (0.6%)	0 (0%)	5.03 [0.24 to 104.3]	+0.6 [-0.2 to 1.4]
Gastrointestinal Bleeding	2 (0.6%)	0 (0%)	5.03 [0.24 to 104.3]	+0.6 [-0.2 to 1.3]

Other Major Bleeding	1 (0.3%)	0 (0%)	3.02 [0.12 to 73.8]	+0.3 [-0.3 to 0.9]
Fatal Bleeding	0 (0%)	0 (0%)	—	—
Clinically Relevant Non-Major Bleeding	5 (1.5%)	3 (0.9%)	1.68 [0.40 to 6.98]	+0.5 [-1.1 to +2.2]

OCEAN TRIAL, 2025	Rivaroxaban (n=634)	Aspirin (n=565)	Relative Risk (RR [95% CI])	Absolute Difference (95% CI) (Calculated)
Primary Efficacy Outcome				
Composite: Stroke, systemic embolism, new covert embolic stroke	5 (0.8%)	9 (1.6%)	0.50 [0.17 to 1.47]	-0.8 [-2.0 to 0.4]
All Stroke	5 (0.8%)	7 (1.2%)	0.64 [0.20 to 1.99]	-0.4 [-1.6 to 0.7]
Systemic Embolism	0 (0.0%)	0 (0.0%)	—	—
New Covert Embolic Stroke	0 (0%)	2 (0.4%)	0.18 [0.01 to 3.71]	-0.4 [-1.1 to 0.2]
TIA	1 (0.2%)	3 (0.5%)	0.30 [0.03 to 2.85]	-0.4 [-1.1 to 0.3]
All-cause mortality	10 (1.6%)	7 (1.2%)	1.27 [0.49 to 3.32]	+0.3 [-0.9 to 1.6]
Primary Safety Outcome				
Composite: Fatal or Major Bleeding	10 (1.6%)	4 (0.6%)	2.51 [0.79 to 7.95]	+0.9 [-0.2 to 2.1]
Major Bleeding	10 (1.6%)	4 (0.7%)	2.23 [0.70 to 7.06]	+0.9 [-0.3 to 2.1]
Intracranial Bleeding	5 (0.8%)	1 (0.2%)	4.46 [0.52 to 38.03]	+0.6 [-0.2 to 1.4]
Gastrointestinal Bleeding	3 (0.5%)	2 (0.4%)	1.34 [0.22 to 7.97]	+0.1 [-0.7 to 0.9]

**Other Major Bleeding**	**2 (0.3%)**	**1 (0.2%)**	**1.78 [0.16 to 19.60]**	**+0.1 [-0.4 to 0.7]**
**Fatal Bleeding**	**0 (0%)**	**0 (0%)**	**—**	**—**
**Minor Bleeding**	**71 (11.2%)**	**17 (3.0%)**	**3.72 [2.22 to 6.24]**	**+8.2 [5.4 to 11.0]**
**Clinically Relevant Non-Major Bleeding**	**32 (5.1%)**	**8 (1.4%)**	**3.56 [1.66 to 7.67]**	**+3.6 [1.7 to 5.6]**
**Major or Minor Bleeding**	**80 (12.6%)**	**20 (3.5%)**	**3.56 [2.21 to 5.74]**	**+9.1 [6.1 to 12.1]**

CI: confidence interval, OAC: oral anticoagulant, TIA: transient ischemic attack

TH Open | DOI 10.1055/a-2916-8912 | © The Author(s).
